# Trends, pathological classification of renal diseases proved by biopsy: A 10-year retrospective cohort study in an East Chinese Tertiary Center

**DOI:** 10.1097/MD.0000000000048595

**Published:** 2026-05-08

**Authors:** Rui Dong, Jing Sun, Mengnan Guo, Qi Bian, Zhiyong Guo, Jing Xu

**Affiliations:** a Nephrology Department, The PLA Naval Medical University, Shanghai Changhai Hospital, Shanghai, China; b Department of General Medicine, the First Affiliated Hospital of Naval Medical University, Shanghai Changhai Hospital, Shanghai, China.

**Keywords:** China, chronic kidney disease, renal biopsy, spectrum of kidney disease

## Abstract

This study aimed to investigate regional epidemiological trends of chronic kidney disease (CKD) to elucidate their etiologies and inform the development of preventive and diagnostic strategies. We retrospectively analyzed 2100 hospitalized patients undergoing native renal biopsy from January 1, 2012, to December 31, 2021. The clinical presentation, the prevalence of nephropathy revealed by biopsy, the age-distribution differences of main pathological types, and changes in the prevalence of different diseases between period 1 (2012–2016) and period 2 (2017–2021) were further analyzed. Hematuria or proteinuria not within the range of the nephrotic syndrome (NS) (36.7%), NS (32.6%), chronic renal insufficiency (22.3%) and acute kidney injury (3.1%) were the 4 major clinical indications for renal biopsy. Although the proportion of primary glomerulonephritis (primary GN, from 85.0% to 75.9%) has shown a downward trend, it remained the predominant diagnosis, with secondary glomerulonephritis (secondary GN, from 11.2% to 16.5%) ranking second. Combined nephropathy showed a significant increase (from 0.3% to 2.6%) especially the diabetic nephropathy (DN) combined with other kidney diseases (from 0.2% to 1.8%). Among primary GN, IgA nephropathy (IgAN) was the most common type, followed by membranous nephropathy (MN). Although the proportion of IgAN did not change for the general population, among individuals aged 30–65, there was an upward trend. However, for secondary GN, DN increased significantly while lupus nephritis (LN) showed a downward trend. Additionally, there were significant differences in the age-distribution between DN and IgAN, DN and minimal change disease (MCD), IgAN and MN, and MN and MCD. The epidemiological trend of renal diseases has changed significantly over time. Although the proportion of primary GN has shown a downward trend, it remained the predominant component, with secondary GN ranking second, and combined nephropathy showing a significant increase. Among primary GN, IgAN was the most common type in the study, followed by MN. However, for secondary GN, from period 1 to period 2, DN gradually surpassed LN to become the most common disease type.

## 1. Introduction

Chronic kidney disease (CKD) is an increasingly important public health problem as an important contributor to morbidity and mortality from noncommunicable diseases.^[1]^ According to the recent reports, there are 159.8 million people with CKD in China, which is the largest population in Asia.^[2]^ As we all know, renal biopsy is the gold standard for diagnosing many kidney diseases and often guides further management^[3]^ and evaluation of prognosis. Histopathologic information provided by renal biopsy classified CKD as different pathological types, which constitutes the disease spectrum of kidney diseases. The spectrum of CKD varies a lot in different times, regions and environments.^[4–6]^ In order to explore the prevalence and epidemiological trend of CKD in our center in Shanghai, we retrospectively analyzed 2100 hospitalized patients who underwent native renal biopsy in our center during the past 10 years.

## 2. Methods

This study was approved by the Research Ethics Committee of the First Affiliated Hospital of Naval Medical University (Shanghai Changhai Hospital) (approval number CHEC2021-181). All experiments were performed in accordance with relevant guidelines and regulations. For this retrospective study using existing medical records and pathological data, the requirement for research-specific informed consent was waived by the Research Ethics Committee of the First Affiliated Hospital of Naval Medical University (Shanghai Changhai Hospital).

We conducted a retrospective analysis of medical records of 2100 hospitalized patients who underwent renal biopsies for various indications from 2012 to 2021 at the Nephrology Department of Changhai Hospital in Shanghai, China. The reasons for renal biopsy included hematuria or proteinuria not within the range of the nephrotic syndrome (NS), NS, chronic renal insufficiency (CRI), and acute kidney injury (AKI).

As a routine clinical procedure, written informed consent for the renal biopsy operation was obtained from all patients before the procedure. In our study, all patients underwent clinical assessment, laboratory investigations, and renal biopsy. All biopsies were conducted under ultrasound guidance by a nephrologist, assisted by a qualified ultrasound doctor. The criteria for all kidney biopsies were consistent.

To facilitate analysis, the histological diagnoses were classified into 6 major categories: primary glomerulonephritis (primary GN), secondary glomerulonephritis (secondary GN), acute and chronic tubulointerstitial nephritis (TIN), vascular kidney disease (VD), and hereditary and congenital renal disease (HCRD), as well as combined nephropathy. The specific diseases within each category were as follows:

Primary GN excluded secondary factors and included, but was not limited to, idiopathic membranous nephropathy (MN), IgA nephropathy (IgAN), minimal change disease (MCD), and focal segmental glomerulosclerosis (FSGS).

Secondary GN included, but was not limited to lupus nephritis (LN), Henöch–Schönlein purpura nephritis (HSPN), and DN.

TIN included, but was not limited to acute tubular necrosis, chronic interstitial nephritis.

VD included hypertensive nephrosclerosis (HTN), thrombotic microangiopathy, and ischemic nephropathy.

HCRD included Alport syndrome, Fabry disease, familial recurrent hematuria syndrome, and other hereditary glomerulopathies.

The combined nephropathy included diabetic nephropathy (DN) or non-DN combined with other kidney diseases. DN combined with other kidney diseases included primary GN or another secondary GN. Non-DN combined with other kidney disease included MN combined with IgAN. (Combined nephropathy was strictly defined as the simultaneous presence of two or more independent renal pathologies, where the diagnostic criteria for each disease were fully met based on a comprehensive evaluation of renal tissue using light microscopy, electron microscopy, and immunofluorescence staining. A paradigmatic example is the co-diagnosis of DN and IgAN. The diagnosis of DN, particularly when distinguishing it from nondiabetic renal disease (NDRD), was confirmed using an integrated clinicopathological approach. This required not only the presence of characteristic histopathological lesions (e.g., glomerular hypertrophy, basement membrane thickening, mesangial expansion, Kimmelstiel-Wilson nodules) according to the 2010 Renal Pathology Society classification,^[7]^ but also by correlation with the clinical context, including the duration of the patient diabetes, blood glucose control, proteinuria, the presence of other diabetic microvascular lesions (such as diabetic retinopathy), while excluding other potential causes of kidney injury. Concurrently, the diagnosis of IgAN was independently confirmed by the predominant mesangial deposition of IgA immune complexes.^[8]^ Another typical example is the co-diagnosis of MN and IgAN. MN is caused by the granular deposition of immunoglobulins (mainly IgG) and complement components (mainly C3) along the walls of glomerular capillaries. Electron microscopy confirmed that the electron-dense substances deposited on the epithelial cell side of the glomerular basement membrane (i.e., subepithelial),^[9]^ which is different from IgAN, which is mainly the granular or massive deposition of IgA immune complexes in the mesangial area.^[8]^) (Supplementary materials-pathological criteria.)

In this study, we divided the population into 3 groups based on age: aged 30 and under (young population), 30 < age ≤ 65 (middle-aged group), and aged above 65 years (retired population) for more targeted analysis. This classification aims to avoid misleading conclusions due to an insufficient number of cases in certain age intervals caused by overly detailed grouping. Similarly, we divided the study period into 2 equal time periods, 2012 to 2016 and 2017 to 2021, to eliminate the potential issue of insufficient case numbers in some time periods that could arise from year-by-year grouping, thereby ensuring the accuracy of our conclusions. In addition, we conducted a detailed year-by-year analysis of some key pathological types to further investigate their trends (Supplementary Figures 1s3s4s5s6s6).

No missing data were identified in the dataset; therefore, no imputation was necessary.

### 2.1. Statistical analysis

Categorical variables were presented as percentage with their corresponding 95% confidence intervals (CIs), and continuous variables were presented using the median value along with the interquartile range (IQR). To compare the categorical variables, Pearson χ^2^ test was employed and the Fisher exact test was conducted if any expected frequency of categorical variables was <5. For the continuous variable, we used Mann-Whitney U or Kruskal-Wallis tests (The study’s continuous variable, age, was found to deviate from a normal distribution as per the Kolmogorov–Smirnov test. Bonferroni correction was used to control family-wise error rate). All analyses were performed with SPSS version 25 (IBM Corp, Armonk, NY, USA) and GraphPad (GraphPad Prism 8.0.0, GraphPad Software, San Diego, CA, USA). Statistical significance was set at *P* < .05, and all tests were 2-tailed.

## 3. Results

### 3.1. Demographic features

Among 2100 patients enrolled in this study between January 1, 2012 and December 31, 2021, 986 cases were enrolled from 2012 to 2016 and 1114 cases from 2017 to 2021. The number of renal biopsies increased almost every year in the past 10 years, but the proportion of renal biopsies in inpatients was stable, ranging from 9.0% to 10.4%.

The age at the time of renal biopsy ranged from 18 to 84 years, with a median of 45 (IQR 33, 57) years. Of the full cohort, 20.0% (419) were aged 30 and under, 70.8% (1486) were in the 30 to 65 years age group (30 < age ≤ 65), and 9.3% (195 cases) were aged above 65 years. From period 1 to period 2, the proportion of patients over 65 increased significantly from 6.7% to 11.6% (χ^2^ = 14.825, *P* < .001). Besides, in the whole group there were 1155 (55.0%) male samples, resulting in a male-to-female ratio of 1.22:1, and the gender difference was most pronounced in the elderly patients, with a ratio of 1.83:1.

### 3.2. Clinical features

**Figure 1.**
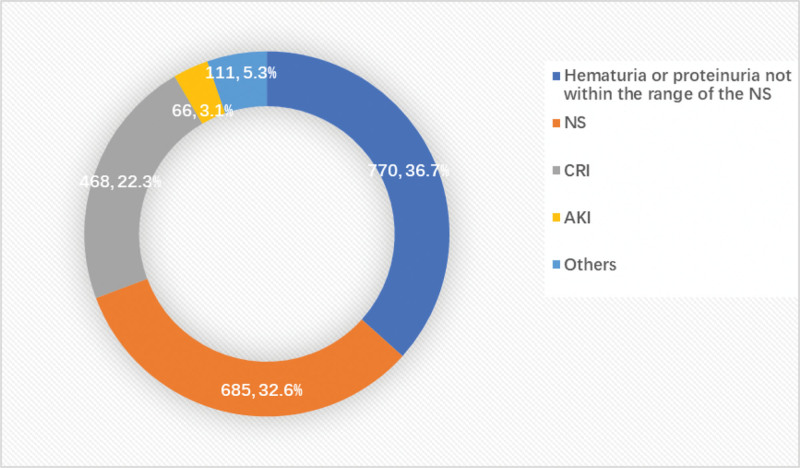
The common clinical indications of renal biopsy in our 2100 patients. NS, nephrotic syndrome; CRI, chronic renal insufficiency; AKI, acute kidney injury.

There were 4 major clinical indications of renal biopsy in the study including hematuria or proteinuria not within the range of NS (770 cases, 36.7%, 95% CI: 34.6–38.7%), NS (685, 32.6%, 95% CI: 30.6–34.6%), CRI (468, 22.3%, 95% CI: 20.5–24.1%), and AKI (66, 3.1%, 95% CI: 2.5–4.0%) (Fig. 1).

### 3.3. Pathology diagnosis

**Figure 2.**
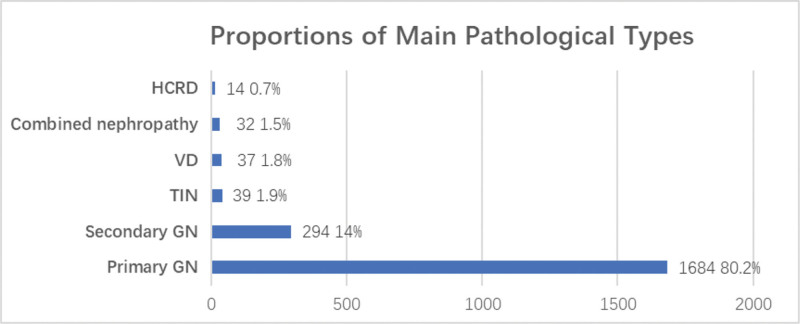
The distributions of pathological diagnosis based on the classification of primary GN, secondary GN, TIN, VD, HCRD, and combined nephropathy in the whole cohort (n = 2100). Primary GN, primary glomerulonephritis; Secondary GN, secondary glomerulonephritis; TIN, acute and chronic tubulointerstitial nephritis; VD, vascular kidney disease; HCRD, hereditary and congenital renal disease.

For all the biopsied cases, primary GN accounted for 80.2% (1684, 95% CI: 78.4–81.9%), secondary GN was 14.0% (294, 95% CI: 12.6–15.5%), TIN 1.9% (39, 95% CI: 1.3–2.5%), VD 1.8% (37, 95% CI: 1.3–2.4%), combined nephropathy 1.5% (32, 95% CI: 1.1–2.1%), and HCRD 0.7% (14, 95% CI: 0.4–1.1%) (Fig. 2).

**Figure 3.**
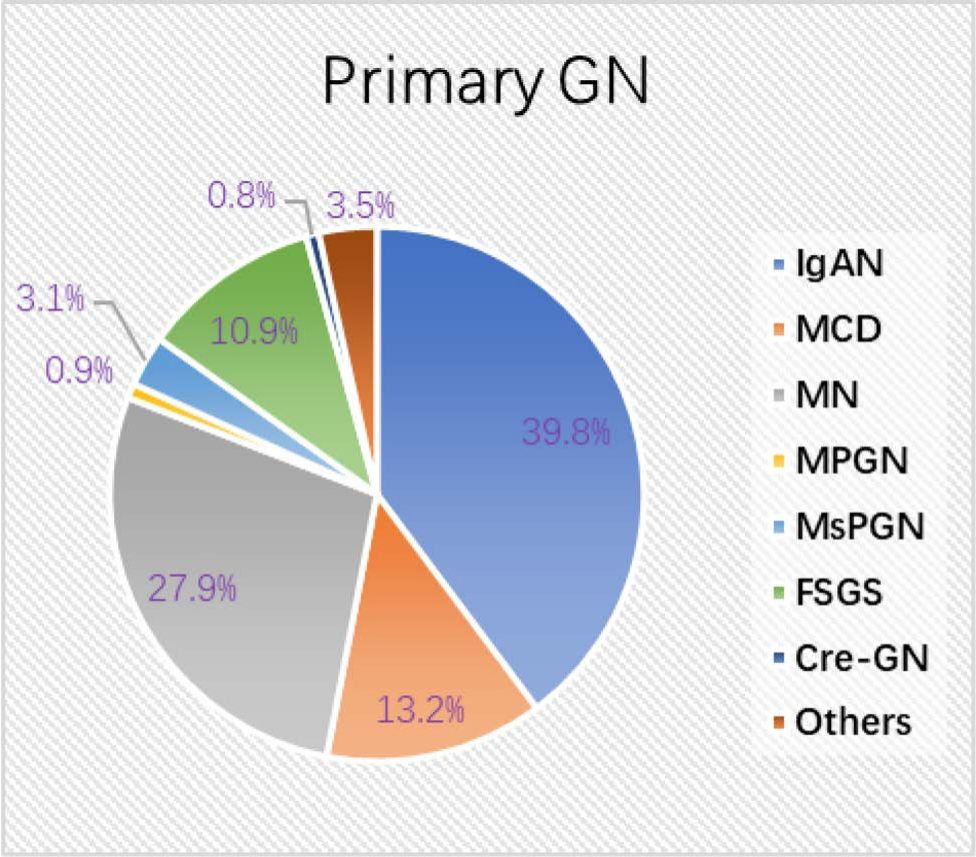
Distribution of the main pathological classifications in primary GN (n = 1684). Primary GN, primary glomerulonephritis; IgAN, IgA nephropathy; MCD, minimal change disease; MN, membranous nephropathy; MPGN, membranoproliferative glomerulonephritis; MsPGN, mesangial proliferative glomerulonephritis; FSGS, focal segmental glomerulosclerosis; Cre-GN, crescentic glomerulonephritis.

Among primary GN, IgAN (670, 39.8%, 95% CI: 37.5–42.1%) and MN (469, 27.9%, 95% CI: 25.7–30.0%) were the most common ones, followed by MCD (13.2%, 223, 95% CI: 11.6–14.9%) (Fig. 3).

**Figure 4.**
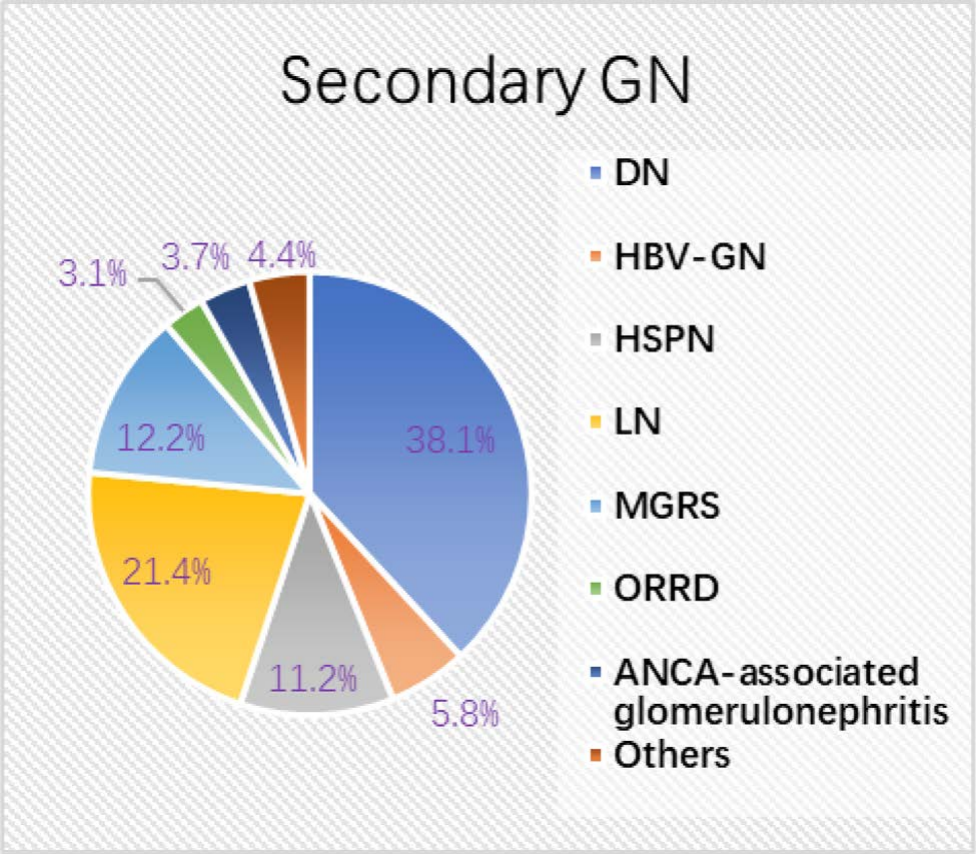
Distribution of the main pathological classifications in secondary GN (n = 294). Secondary GN, secondary glomerulonephritis; DN, diabetic nephropathy; HBV-GN, hepatitis B virus-associated glomerulonephritis; HSPN, Henöch–Schönlein purpura nephritis; LN, lupus nephritis; MGRS, monoclonal gammopathy of renal significance; ORRD, obesity-related renal disease; ANCA, antineutrophil cytoplasmic antibody.

Among secondary GN, DN (112, 38.1%, 95% CI: 32.7–43.7%) and LN (63, 21.4%, 95% CI: 17.0–26.4%) were the most common types, followed by MGRS (36, 12.2%, 95% CI: 8.9–16.4%) and HSPN (33, 11.2%, 95% CI: 8.0–15.2%) (Fig. 4).

### 3.4. Age distribution

Primary GN patients had a median age of 43 (IQR 32, 55) while secondary GN was 50 (IQR 37, 61), there were significant differences between them (Z = 5.167, *P* < .001).

Among primary GN, the median age of patients with IgAN was 39 (IQR 30, 47), MCD was 41 (IQR 27, 56), MN was 53 (IQR 39, 62), and FSGS was 45 (IQR 34, 55), while for secondary GN, DN was 53 (IQR 47, 63), and LN was 34 (IQR 23, 47).

**Figure 5.**
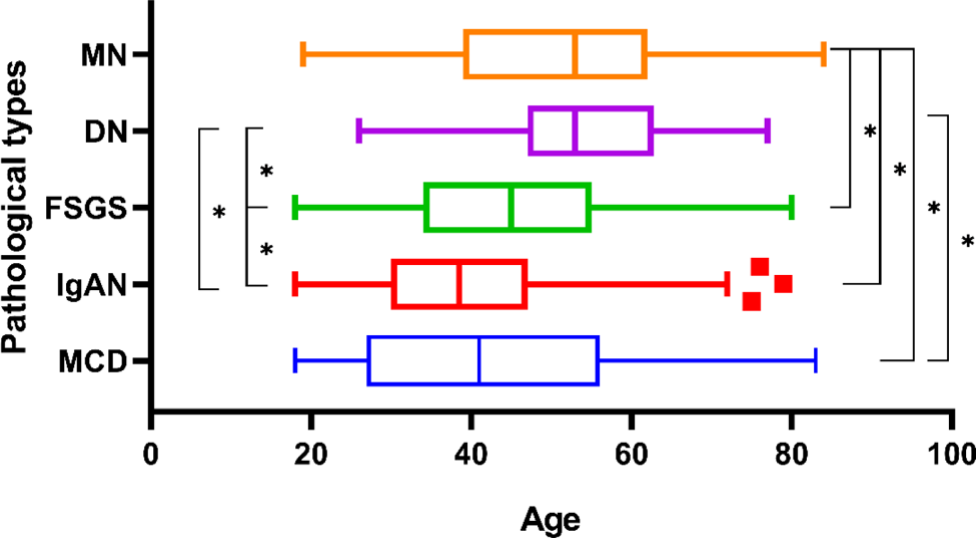
Age-distribution differences among different pathological types. Comparison of age distribution among patients with the 5 major pathological types. *P value < .005 is considered to be statistically significant. P values (Bonferroni correction) = Initial p/10. MN, membranous nephropathy; DN, diabetic nephropathy; FSGS, focal segmental glomerulosclerosis; IgAN, IgA nephropathy; MCD, minimal change disease.

We compared the age-distribution of the main types of primary and secondary GN, and found significant differences in the age-distribution between DN and IgAN (H = 10.082, *P* < .0001) (*P* values (Bonferroni correction) = Initial p/10), DN and FSGS (H = 5.398, *P* < .0001), DN and MCD (H = 7.273, *P* < .0001), IgAN and MN (H = 13.316, *P* < .0001), IgAN and FSGS (H = 4.575, *P* < .0001), MN and FSGS (H = 4.820, *P* < .0001), and MN and MCD (H = 7.554, *P* < .0001). However, there was no difference in age-distribution between MN and DN (H = 2.164, *P* = .305), IgAN and MCD (H = 2.045, *P* = .162), and MCD and FSGS (H = 1.956, *P* = .504) (Fig. 5).

### 3.5. Trends of renal histopathologic diagnosis in the past decade

We further divided the entire study into 2 periods, period 1, from 2012 to 2016 and period 2, from 2017 to 2021. According to the different age at renal biopsy, the subjects were classified into 3 categories: group 1 (aged ≤ 30 years), group 2 (30 years < aged ≤ 65 years), and group 3 (aged > 65 years).

From period 1 to period 2, primary GN was still the largest pathological type, followed by secondary GN, but primary GN had a downward trend (from 85.0% to 75.9%) while secondary GN increased (from 11.2% to 16.5%).

Over the past decade, IgAN and MN were still the primary 2 types of primary GN, and their proportion had not tended to change. However, at the age section of group 2, IgAN tended to rise from 28.8% (197) to 34.6% (278) (χ^2^ = 5.664, *P* = .017), while the MN decreased from 26.2% (179) to 21.8% (175) (χ^2^ = 3.964, *P* = .046).

In secondary GN, the proportion of LN decreased from 3.8% (37) to 2.3% (26), but with no significance, while the proportion of DN increased from 2.6% (26) to 7.7% (86) (χ^2^ = 26.766, *P* < .001) and surpassed LN as the most common type of secondary GN in period 2 (Table 1).

**Table 1:** Trend in the changing proportion of the main pathological types (based on age).

	2012–2016	2017–2021	χ^2^	*P* values
Age ≤ 30 yr, % (95% CI)				
IgAN	92 (38.8, 32.8–45.1)	83 (45.6, 38.5–52.9)	1.949	.163
MN	23 (9.7, 6.4–14.0)	23 (12.6, 8.4–18.0)	0.906	.341
MCD	43 (18.1, 13.6–23.4)	28 (15.4, 10.7–21.2)	0.557	.456
MsPGN	11 (4.6, 2.5–7.9)	2 (1.1, 0.2–3.5)	4.297	.038
FSGS	23 (9.7, 6.4–14.0)	11 (6.0, 3.3–10.2)	1.850	.174
LN	17 (7.2, 4.4–11.0)	7 (3.8, 1.7–7.4)	2.110	.146
DN	2 (0.8, 0.2–2.7)	2 (1.1, 0.2–3.5)		1.000*
DN+	0 (0)	0 (0)		
30 yr < Age ≤ 65 yr, % (95% CI)				
IgAN	197 (28.8, 25.5–32.3)	278 (34.6, 31.4–38.0)	5.664	.017
MN	179 (26.2, 23.0–29.6)	175 (21.8, 19.0–24.7)	3.964	.046
MCD	73 (10.7, 8.5–13.2)	58 (7.2, 5.6–9.2)	5.513	.019
MsPGN	20 (2.9, 1.9–4.4)	12 (1.5, 0.8–2.5)	3.601	.058
FSGS	72 (10.5, 8.4–13.0)	68 (8.5, 6.7–10.5)	1.859	.173
LN	18 (2.6, 1.6–4.0)	16 (2.0, 1.2–3.1)	0.682	.409
DN	22 (3.2, 2.1–4.7)	66 (8.2, 6.5–10.3)	16.549	<.001
DN+	1 (0.1, 0.0–0.7)	16 (2.0, 1.2–3.2)	11.122	.001
Age > 65 yr, %(95% CI)				
IgAN	7 (10.6, 4.9–19.7)	13 (10.1, 5.8–16.2)	0.013	.908
MN	27 (40.9, 29.6–53.0)	42 (32.6, 24.9–41.0)	1.332	.248
MCD	6 (9.1, 3.9–17.8)	14 (10.9, 6.4–17.1)	0.147	.701
MsPGN	6 (9.1, 3.9–17.8)	1 (0.8, 0.1–3.6)	8.724	.003
FSGS	1 (1.5, 0.2–6.9)	8 (6.2, 3.0–11.4)	2.178	.140
LN	2 (3.0, 0.6–9.4)	3 (2.3, 0.7–6.1)		1.000*
DN	2 (3.0, 0.6–9.4)	18 (14.0, 8.8–20.7)	5.660	.017
DN+	1 (1.5, 0.2–6.9)	4 (3.1, 1.1–7.2)	0.034	.664*
All age sections, %(95% CI)				
Primary GN	838 (85.0, 82.7–87.1)	846 (75.9, 73.4–78.4)	26.952	<.001
Secondary GN	110 (11.2, 9.3–13.2)	184 (16.5, 14.4–18.8)	12.485	<.001
Combined Nephropathy	3 (0.3, 0.1–0.8)	29 (2.6, 1.8–3.7)	18.422	<.001
IgAN	296 (30.0, 27.2–32.9	374 (33.6, 30.8–36.4)	3.038	.081
MN	229 (23.2, 20.7–25.9)	240 (21.5, 19.2–24.0)	0.852	.356
MCD	122 (12.4, 10.4–14.5)	100 (9.0, 7.4–10.8)	6.383	.012
MsPGN	37 (3.8, 2.7–5.1)	15 (1.3, 0.8–2.2)	12.539	<.001
FSGS	96 (9.7, 8.0–11.7)	87 (7.8, 6.3–9.5)	2.441	.118
LN	37 (3.8, 2.7–5.1)	26 (2.3, 1.6–3.3)	3.617	.057
DN	26 (2.6, 1.8–3.8)	86 (7.7, 6.3–9.4)	26.766	<.001
DN+	2 (0.2, 0.0–0.6)	20 (1.8, 1.1–2.7)	12.796	<.001
Total	986 (100%)	1114 (100%)		

* Analyzed by Fisher’s exact test. CI = confidence interval, DN = diabetic nephropathy, DN+ = diabetic nephropathy-based combined nephropathy, FSGS = focal segmental glomerulosclerosis, IgAN = IgA nephropathy, LN = lupus nephritis, MCD = minimal change disease, MN = membranous nephropathy, MsPGN = mesangial proliferative glomerulonephritis, Primary GN = primary glomerulonephritis, Secondary GN = secondary glomerulonephritis.

IgAN =.

**Figure 6.**
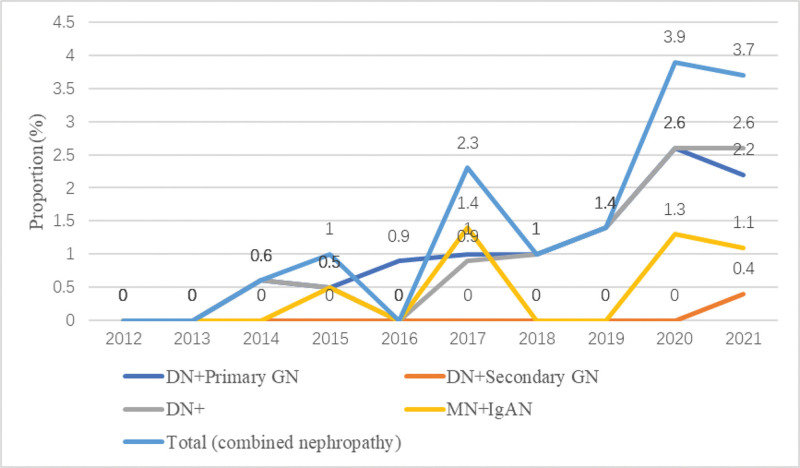
The annual proportion of combined nephropathy based on DN compared to that of MN combined with IgAN. Annual proportion of pathological subtypes in combined nephropathy: a comparison of diabetic (DN + Primary GN, DN + Secondary GN) vs Non-Diabetic (MN + IgAN) groups. DN + Primary GN, diabetic nephropathy combined with primary glomerulonephritis; DN + Secondary GN, diabetic nephropathy combined with secondary glomerulonephritis; DN+, diabetic nephropathy-based combined nephropathy; MN + IgAN, membranous nephropathy combined with IgA nephropathy.

In addition, the proportion of combined nephropathy increased from 0.0% (0) in 2012 to 3.7% (10) in 2021. The DN combined with primary GN accounted for 65.6% (21), followed by MN combined with IgAN 31.3% (10), and DN combined with another secondary GN 3.1% (1). From period 1 to period 2, the proportion of DN combined with other kidney disease increased gradually, from 0.2% (2) to 1.8% (20) (χ^2^ = 12.796, *P* < .001), and was far higher than those of the other combined kidney diseases since 2018 (Fig. 6).

## 4. Discussion

We performed a retrospective analysis of 2100 patients who received renal biopsies from 2012 to 2021 at the Nephrology Department of Changhai Hospital in Shanghai, China, and investigated the clinical manifestation, the proportion and trends of the common pathological types, and the age-distribution of major diseases. Our findings revealed a significant shift in the spectrum of renal diseases over the past decade.

Hematuria or proteinuria not within the range of NS was the main clinical feature of our study population (36.7%), followed by NS (32.6%), CRI (22.3%), and AKI (3.1%) which differs from the findings reported by Chen-Wei Yen et al, where acute nephritis associated with autoimmune disorders was the primary indication for renal biopsy (55.7%).^[10]^

According to the age-distribution data, patients over 65 constituted a relatively small proportion (9.3%), with a male predominance. However, from period 1 to period 2, the proportion of over 65 patients who underwent the procedure increased, which is in line with the reported prevalence of kidney disease in the elderly population in Nanjing.^[11]^ This trend may be attributed to the aging of the Chinese population, improved biopsy technology expanding the applicable age range, enhanced disease awareness via health education, and better economic conditions. The male predominance in elderly biopsy patients is also consistent with previous reports.^[12,13]^ Further research is needed to determine the reasons for this discrepancy, as well as potential gender differences in risk factors, outcomes, and treatment of kidney disease.

To delve deeper into the age-distribution of various pathological classifications, we conducted an additional analysis. The results showed that IgAN and LN were more likely to occur in young people with the median ages of 39 and 34 years, respectively, while MN and DN were more common in the elderly, both with a median age of 53 years. This is consistent with previous reports.^[13]^ To further evaluate the diagnostic potential of age, we analyzed the age distributions of common glomerular diseases. Our analysis revealed 2 distinct patterns. First, for several disease pairs (e.g., IgAN vs MN, IgAN vs FSGS, DN vs FSGS), the age distributions were significantly different (*P* < .0001), indicating that age is a valuable parameter for their clinical distinction. Second, for other pairs with high prevalence, such as MN vs DN and IgAN vs MCD, no statistically significant difference in age distribution was observed. These diseases are similar in clinical presentation, and the nondifferential age distribution makes their clinical differentiation more difficult. Therefore, a renal biopsy is needed for a definitive diagnosis.

Primary GN remained the predominant renal disease in our cohort over the decade (80.2%), consistent with reports from other Chinese centers (61.7% to 82.7%).^[14–16]^ However, from period 1 to period 2, the proportion of primary GN showed a significant downward trend from 85.0% to 75.9%, in accord with an analysis by Minghui Zhao et al.^[17]^ In contrast, the proportion of secondary GN increased significantly from 11.2% to 16.5% which aligns with the previous report.^[18]^

In primary GN, among patients of all ages, IgAN ranked first in both time periods, followed by MN, and the changes in the proportion of the 2 were not statistically significant. However, in elderly patients, the incidence of MN is significantly higher than that of IgAN, which is consistent with our conclusion that the age-distribution of different pathological types is different. Previous studies have shown different trends in the incidence of IgAN, which may be related to the different indications of renal biopsy patients and regional differences.^[19–21]^ Although the overall proportion of IgAN did not change significantly with age, a notable increase was observed in adults aged 30 to 65 years, indicating age-related differences in incidence.

MN was the second most prevalent primary GN (22.3% of all biopsy patients) in our study, higher than the 12.6% reported in a prior Chinese study,^[20]^ but similar to the 20% prevalence in Brazil.^[22]^ From period 1 to period 2, the proportion of biopsy-proven MN decreased overall, although the difference was not statistically significant. This trend was mostly due to the clinical use of tests for MN antibodies, notably the detection of blood phospholipase A2 receptor (PLA2R) antibody levels. Meta-analysis has demonstrated the sensitivity of the serum anti-PLA2R antibody to be 78% and its specificity to be 99% in diagnosing primary MN,^[23]^ making it an effective tool for primary MN detection. This test is less invasive and easier to perform than renal biopsy, enabling clinical diagnosis of MN, and reducing the number of biopsy procedures. Therefore, although the proportion of MN derived from renal biopsy data did not significantly decrease, this does not mean that the overall incidence of MN did not increase. In our study, the proportion of MN showed an age-dependent increase, accounting for 11.0% in patients aged 30 and under, 23.8% in those aged 30 to 65, and 35.4% in patients over 65. In contrast, the proportion of IgAN in patients over 65 was found to be comparatively lower, comprising 10.3% of the cases. This finding is consistent with other studies that identify MN as the most prevalent kidney disease in older adults.^[12,13,24]^ Another study on renal biopsy spectrum in elderly Chinese patients showed that 53.38% of the primary GN in the elderly were MN, which aligns with our data 51.5%.^[11]^ This age-related prevalence of MN could be attributed to a combination of genetic factors, immune system changes, and environmental exposure as people aged.^[7]^

Among secondary GN, DN accounted for 38.1% of all diagnoses, followed by LN (21.4%). LN was the most prevalent secondary GN in the people aged 30 and under, while DN was the most common one in the elderly. Although the difference was not statistically significant, LN accounted for the highest proportion of secondary GN in period 1 but fell to second place in period 2. In contrast, the proportion of DN increased significantly, surpassing LN to become the most common type of secondary GN in period 2. This is consistent with a previous report, which showed that the proportion of LN in secondary GN decreased significantly, from 54.7% during 1979 to 2002 to 32.9% during 2003 to 2014.^[18]^ The decrease in LN may be related to the advancements in medical practices. The popularity of autoantibody testing, the training of specialists, and new treatment guidelines have all facilitated the early identification and intervention in systemic lupus erythematosus (SLE).^[25]^ Furthermore, the evaluation of kidney involvement in SLE patients has become more rigorous, and the use of new biologics such as rituximab and belimumab can effectively prevent kidney injury, which significantly improves the clinical prognosis of SLE and reduces the incidence of LN.^[26,27]^ As for the upward trend in DN, this may be related to the increased incidence of obesity, prediabetes, and diabetes mellitus (DM). In 2008, DM and prediabetes prevalence rates were 9.7% and 15.5%, respectively.^[28]^ As of 2020, a meta-analysis reported that the overall DN prevalence in China was 21.8%,^[29]^ and another study showed that the number of people with DM in China could reach 142 million by the year 2035.^[30]^ In addition, a study by Else van den Berg et al found a positive association between a Westernized diet and DN, suggesting that the increasing prevalence of DN may be partly attributed to lifestyle factors.^[31]^ Notably, the proportion of combined nephropathy increased in our cohort, with DN-based combined nephropathy accounting for the largest share and showing the fastest growth (0.2% in 2012–2016 to 1.8% in 2017–2021). This trend was consistent with the increase in the proportion of DN. But a history of DM alone does not always lead to a diagnosis of DN. Previously, DN was diagnosed clinically, but subsequent studies revealed inconsistencies between clinical and pathological findings. Data from Shanghai Ruijin Hospital indicate that 24.6% of diabetic patients diagnosed with DN, 68.6% with NDRD, and 6.8% with DN + NDRD.^[32]^ This indicates that the nephropathy complicated with DM is not completely DN. This discrepancy may be due to the insufficient evidence available for clinical diagnosis of DN, relying solely on the urinary albumin to creatinine ratio, assessment of glomerular filtration rate, and history of DM. However, it is important to emphasize that some comorbidities of DM, such as obesity and hypertension, can independently cause kidney damage, complicating the determination of the cause of kidney disease in DM patients. With the development of renal biopsy technology, more and more patients with DM and kidney disease can be accurately classified as having DN, NDRD, or a combination of both.^[33]^ Therefore, renal biopsy becomes an indispensable procedure for a definitive diagnosis. In addition, we observed that although the proportion of DN and DN-based combined nephropathy increased significantly across the entire patient cohort, this trend was only evident in patients aged 30 to 65. In contrast, among those over 65, only DN showed an upward trend while DN-based combined nephropathy did not. This may suggest that younger DN patients are more likely to develop other types of kidney disease and imply that kidney biopsy is of greater significance for the definitive diagnosis of kidney disease in younger patients with DN.

## 5. Conclusion

The spectrum of renal diseases in a single center in Shanghai has experienced significant changes in the past decade, similar to other areas in China. Primary GN remained the most commonly diagnosed cause, but the proportion of secondary GN was gradually increasing. And the proportion of combined nephropathy has increased. Among primary GN, IgAN and MN were the 2 most frequent diagnoses. And in the 30 to 65 years age group, there was a clear upward trend in IgAN. However, for secondary GN, although DN and LN have always been the 2 diagnostic types with the largest proportion, DN surpassed LN to become the first one from period 1 to period 2. Furthermore, there has been a dramatic increase in combined nephropathy, especially the DN-based combined nephropathy. And for age-distribution, there was no significant difference between MN and DN, IgAN and MCD, and MCD and FSGS. Having an understanding of the changes in the spectrum of kidney diseases and the prevalence of different types of kidney diseases in different gender and age can provide effective guidance for the prevention and treatment of kidney diseases. It also highlights the need to consider individual patient characteristics when diagnosing and managing kidney diseases, and to explore novel treatment approaches with close monitoring of therapeutic efficacy throughout the clinical course.

## Author contributions

**Conceptualization:** Rui Dong, Zhiyong Guo, Jing Xu.

**Data curation:** Jing Sun, Mengnan Guo.

**Formal analysis:** Zhiyong Guo.

**Investigation:** Rui Dong, Qi Bian.

**Methodology:** Jing Sun.

**Project administration:** Zhiyong Guo.

**Resources:** Qi Bian, Jing Xu.

**Software:** Rui Dong, Mengnan Guo, Qi Bian.

**Writing – original draft:** Jing Sun, Mengnan Guo.

**Writing – review & editing:** Rui Dong, Jing Xu.















## References

[1] Obrador GT Levin A. CKD hotspots: challenges and areas of opportunity. Semin Nephrol. 2019;39:308–14. 31054631 10.1016/j.semnephrol.2019.02.009

[2] Liyanage T Toyama T Hockham C. Prevalence of chronic kidney disease in Asia: a systematic review and analysis. BMJ Glob Health. 2022;7:e007525. 10.1136/bmjgh-2021-007525 PMC8796212 35078812

[3] Kitterer D Gürzing K Segerer S. Diagnostic impact of percutaneous renal biopsy. Clin Nephrol. 2015;84:311–22. 26396098 10.5414/CN108591

[4] Covic A Vlad CE Căruntu ID. Epidemiology of biopsy-proven glomerulonephritis in the past 25 years in the North-Eastern area of Romania. Int Urol Nephrol. 2022;54:365–76. 33991297 10.1007/s11255-021-02881-z

[5] Molnár A Thomas MJ Fintha A. Kidney biopsy-based epidemiologic analysis shows growing biopsy rate among the elderly. Sci Rep. 2021;11:24479. 34966177 10.1038/s41598-021-04274-9 PMC8716536

[6] Mohapatra A Kakde S Annapandian VM. Spectrum of biopsy proven renal disease in South Asian children: two decades at a tropical tertiary care centre. Nephrology (Carlton). 2018;23:1013–22. 28846194 10.1111/nep.13160 PMC7615900

[7] Tervaert TW Mooyaart AL Amann K; Renal Pathology Society. Pathologic classification of diabetic nephropathy. J Am Soc Nephrol. 2010;21:556–63. 20167701 10.1681/ASN.2010010010

[8] Trimarchi H Barratt J Cattran DC. Oxford classification of IgA nephropathy 2016: an update from the IgA Nephropathy Classification Working Group. Kidney Int. 2017;91:1014–21. 28341274 10.1016/j.kint.2017.02.003

[9] Fogo AB Lusco MA Najafian B Alpers CE. AJKD Atlas of renal pathology: membranous nephropathy. Am J Kidney Dis. 2015;66:e15–7. 26300203 10.1053/j.ajkd.2015.07.006

[10] Yen CW Chen TD Yen TH Yu MC. The pathological spectrum of pediatric kidney disease: 18-year experience from a single tertiary care center in Northern Taiwan. Pediatr Neonatol. 2023;64:26–31. 36163129 10.1016/j.pedneo.2022.07.005

[11] Jin B Zeng C Ge Y. The spectrum of biopsy-proven kidney diseases in elderly Chinese patients. Nephrol Dial Transplant. 2014;29:2251–9. 25034755 10.1093/ndt/gfu239

[12] Brown CM Scheven L O’Kelly P Dorman AM Walshe JJ. Renal histology in the elderly: indications and outcomes. J Nephrol. 2012;25:240–4. 21725922 10.5301/JN.2011.8447

[13] Yokoyama H Sugiyama H Sato H. Renal disease in the elderly and the very elderly Japanese: analysis of the Japan Renal Biopsy Registry (J-RBR). Clin Exp Nephrol. 2012;16:903–20. 23053590 10.1007/s10157-012-0673-8

[14] Li LS Liu ZH. Epidemiologic data of renal diseases from a single unit in China: analysis based on 13,519 renal biopsies. Kidney Int. 2004;66:920–3. 15327382 10.1111/j.1523-1755.2004.00837.x

[15] Zhang X Liu S Tang L. Analysis of pathological data of renal biopsy at one single center in China from 1987 to 2012. Chin Med J (Engl). 2014;127:1715–20. 24791880

[16] Pan X Xu J Ren H. Changing spectrum of biopsy-proven primary glomerular diseases over the past 15 years: a single-center study in China. Contrib Nephrol. 2013;181:22–30. 23689564 10.1159/000348638

[17] Li J Cui Z Long J. Primary glomerular nephropathy among hospitalized patients in a national database in China. Nephrol Dial Transplant. 2018;33:2173–81. 29509919 10.1093/ndt/gfy022

[18] Hou JH Zhu HX Zhou ML. Changes in the spectrum of kidney diseases: an analysis of 40,759 biopsy-proven cases from 2003 to 2014 in China. Kidney Dis (Basel). 2018;4:10–9. 29594138 10.1159/000484717 PMC5848489

[19] Gül CB Küçük M Öztürk S. Trends of primary glomerular disease in Turkey: TSN-GOLD registry report. Int Urol Nephrol. 2022;54:2285–94. 35107695 10.1007/s11255-022-03123-6

[20] Yang Y Zhang Z Zhuo L Chen DP Li WG. The spectrum of biopsy-proven glomerular disease in China: a systematic review. Chin Med J (Engl). 2018;131:731–5. 29521297 10.4103/0366-6999.226906 PMC5865320

[21] Li H Yu X Lan P. Spectrum of biopsy-proven kidney diseases in northwest China: a review of 30 years of experiences. Int Urol Nephrol. 2022;54:2609–16. 35286580 10.1007/s11255-022-03168-7

[22] Polito MG de Moura LA Kirsztajn GM. An overview on frequency of renal biopsy diagnosis in Brazil: clinical and pathological patterns based on 9,617 native kidney biopsies. Nephrol Dial Transplant. 2010;25:490–6. 19633091 10.1093/ndt/gfp355

[23] Du Y Li J He F. The diagnosis accuracy of PLA2R-AB in the diagnosis of idiopathic membranous nephropathy: a meta-analysis. PLoS One. 2014;9:e104936. 25136841 10.1371/journal.pone.0104936 PMC4138154

[24] Verde E Quiroga B Rivera F López-Gómez JM. Renal biopsy in very elderly patients: data from the Spanish Registry of Glomerulonephritis. Am J Nephrol. 2012;35:230–7. 22343659 10.1159/000336307

[25] Tedeschi SK Johnson SR Boumpas DT. Multicriteria decision analysis process to develop new classification criteria for systemic lupus erythematosus. Ann Rheum Dis. 2019;78:634–40. 30692164 10.1136/annrheumdis-2018-214685 PMC7057251

[26] Almaani S Meara A Rovin BH. Update on lupus nephritis. Clin J Am Soc Nephrol. 2017;12:825–35. 27821390 10.2215/CJN.05780616 PMC5477208

[27] Alforaih N Whittall-Garcia L Touma Z. A review of lupus nephritis. J Appl Lab Med. 2022;7:1450–67. 35932197 10.1093/jalm/jfac036

[28] Yang SH Dou KF Song WJ. Prevalence of diabetes among men and women in China. N Engl J Med. 2010;362:2425–6; author reply 2426. 20578276

[29] Zhang XX Kong J Yun K. Prevalence of diabetic nephropathy among patients with type 2 diabetes mellitus in China: a meta-analysis of observational studies. J Diabetes Res. 2020;2020:2315607. 32090116 10.1155/2020/2315607 PMC7023800

[30] Zimmet PZ Magliano DJ Herman WH Shaw JE. Diabetes: a 21st century challenge. Lancet Diabetes Endocrinol. 2014;2:56–64. 24622669 10.1016/S2213-8587(13)70112-8

[31] van den Berg E Hospers FA Navis G. Dietary acid load and rapid progression to end-stage renal disease of diabetic nephropathy in Westernized South Asian people. J Nephrol. 2011;24:11–7. 20872351 10.5301/jn.2010.5711

[32] Xu J Hu XF Huang W. The clinicopathological characteristics of diabetic nephropathy and non-diabetic renal diseases in diabetic patients. Zhonghua Nei Ke Za Zhi. 2017;56:924–9. 29202533 10.3760/cma.j.issn.0578-1426.2017.12.007

[33] Anbuselvam B Panneerselvam SR Thoppalan B Kumar M. The prevalence and clinicopathological spectrum of nondiabetic renal disease in patients with diabetes in a tertiary care center. Saudi J Kidney Dis Transpl. 2023;34(Suppl 1):S161–9. 38995284 10.4103/sjkdt.sjkdt_158_22

