# Supplementary material for: Trends, pathological classification of renal diseases proved by biopsy: A 10-year retrospective cohort study in an East Chinese Tertiary Center
Source: Medicine (Baltimore). 2026 May 8;105(19):e48595. doi: 10.1097/MD.0000000000048595 (PMC13166505; doi:10.1097/MD.0000000000048595)
Supplement: Supplementary file 1 [file medi-105-e48595-s001.docx]

**Standards for Diabetic Kidney Disease (DKD)**

The authoritative standard currently widely accepted and implemented globally is the classification standard established by the Renal Pathology Society (RPS) in 2010. This standard aims to more precisely assess the severity of diabetic kidney injury through unified pathological descriptions and classifications, and to establish connections with the patient's clinical manifestations and prognosis.

The following are the core components of the RPS classification standard:

**I. Four Core Pathological Indicators (Lesion Characteristics)**

The RPS classification primarily evaluates the following four key pathological changes:

1. **Mesangial expansion**
   - **Description**: Increase in mesangial matrix and/or proliferation of mesangial cells, which is the earliest lesion of DKD.
   - **Grading**: Classified as mild (a) or severe (b).
     - **Mild (a)**: Widening of the mesangial area not exceeding the diameter of an adjacent peripheral capillary lumen.
     - **Severe (b)**: Widening of the mesangial area exceeding the diameter of an adjacent peripheral capillary lumen.
2. **Nodular sclerosis (Kimmelstiel-Wilson nodules)**
   - **Description**: Round, laminated sclerotic nodules formed in the mesangial area; a characteristic lesion of DKD, but not present in all patients.
   - **Grading**: Only "present" or "absent", no grading.
3. **Glomerular basement membrane (GBM) thickness**
   - **Description**: Observed under electron microscopy, a uniform thickening of the basement membrane of the glomerular capillary walls.
   - **Grading**: Usually assessed by measurement, but the classification focuses mainly on its presence.
4. **Exudative lesions**
   - **Description**: Includes "fibrin caps" and "capsular drops".
     - **Fibrin cap**: Eosinophilic deposits within the glomerular capillary lumen.
     - **Capsular drop**: Eosinophilic drop-like deposits between the Bowman's capsule basement membrane and the parietal epithelial cells.
   - **Grading**: Only "present" or "absent".

**II. RPS Pathological Classification System (Categories)**

Based on the above lesions, the RPS standard classifies glomerular lesions in DKD into the following four categories:

- **Class I**: Isolated GBM thickening
  - Appears essentially normal under light microscopy; diagnosis relies on electron microscopy. Lesions are very mild.
- **Class II**: Mesangial proliferative lesions
  - **Class IIa**: Mild mesangial expansion
  - **Class IIb**: Severe mesangial expansion
  - This is the most common type, reflecting disease progression.
- **Class III**: Nodular sclerosis (Kimmelstiel-Wilson nodules)
  - At least one definite K-W nodule, usually accompanied by Class IIb mesangial expansion. Represents advanced lesions.
- **Class IV**: Advanced sclerotic diabetic nephropathy
  - **Global glomerulosclerosis** affecting more than 50% of glomeruli.
  - This is an end-stage lesion, often overlapping with other classes.

### III. Other Essential Indicators for Evaluation

In addition to the glomerular lesions mentioned above, a complete pathological report must include the following two important assessments, which have a significant impact on prognosis:

1. **Interstitial and Vascular Lesions**
   - **Tubular atrophy** and **Interstitial fibrosis**: Graded by the affected cortical area as mild (<25%), moderate (25-50%), or severe (>50%). The greater the extent, the worse the renal function prognosis.
   - **Arteriosclerosis** and **Arteriolar hyalinosis**: Assess the degree of vascular injury.
2. **Presence of Non-Diabetic Renal Disease**
   - Diabetic patients may also have other concurrent kidney diseases, such as **membranous nephropathy, IgA nephropathy, Focal Segmental Glomerulosclerosis (FSGS),** etc. A key role of pathological biopsy is to differentiate these conditions, as their treatment plans are completely different.

**Standards for IgA Nephropathy Pathological**

The pathological diagnosis and classification standards for IgA Nephropathy (IgAN) have evolved over many years. Currently, there are two main standard systems used globally, applied in different regions and contexts. The current most mainstream and widely adopted standard is the revised 2016 version of the "Oxford Classification" by the International IgA Nephropathy Pathology Working Group in collaboration with the Renal Pathology Society (RPS), commonly referred to as the "Oxford Classification".

Here is a detailed explanation:

### ****I.**** Core Diagnostic Criterion: The observation of granular or lumpy deposits of immunoglobulin, predominantly IgA, in the mesangial area of the glomeruli. This is the "gold standard" for diagnosing IgA Nephropathy.

**Commonly Associated Pathological Features Include:**

1. **Often accompanied by C3 deposition:** Complement C3 co-depositing with IgA is very common.
2. **May be accompanied by IgG or IgM deposition:** But the intensity is usually weaker than that of IgA.
3. **Electron microscopy:** Reveals **electron-dense deposits in the mesangial area,** which is ultrastructural evidence of immune complex deposition. Sometimes, deposits may extend to the peripheral capillary walls.

**Important Note:** The presence of IgA deposits alone is not sufficient for a definitive diagnosis of primary IgA Nephropathy. It must be **combined with t**he patient's clinical presentation (e.g., recurrent macroscopic hematuria, proteinuria) and **the exclusion of o**ther diseases that can cause mesangial IgA deposits (e.g., Henoch-Schönlein purpura, liver cirrhosis, systemic lupus erythematosus, celiac disease).

### ****II. Pathological Classification Standards****

The purpose of pathological classification is to assess the severity of kidney lesions, predict the risk of disease progression, and inform treatment decisions. There are currently two major systems:

#### ****1. The Oxford Classification (MEST-C Score) - The Current International Mainstream Standard****

This classification was first proposed in 2009 and revised in 2016 to add a new scoring item ("C"), forming the current MEST-C scoring system. It is based on four pathological features with high reproducibility and a clear correlation to prognosis:

- **M - Mesangial hypercellularity**
  - **M0:** ≤50% of glomeruli show mesangial hypercellularity (less than 4 cells per mesangial area)
  - **M1:** >50% of glomeruli show mesangial hypercellularity (4 or more cells per mesangial area)
- **E - Endocapillary hypercellularity**
  - **E0:** No endocapillary hypercellularity
  - **E1:** Endocapillary hypercellularity present (capillary lumens narrowed by infiltrating cells)
- **S - Segmental glomerulosclerosis**
  - **S0:** No segmental glomerulosclerosis
  - **S1:** Segmental glomerulosclerosis present (including adhesions)
- **T - Tubular atrophy / Interstitial fibrosis**
  - **T0:** ≤25% of the cortical area involved
  - **T1:** 26-50% of the cortical area involved
  - **T2:** >50% of the cortical area involved (a very strong indicator of poor prognosis)
- **C – Crescents** - Added in the 2016 revision
  - **C0:** No crescents
  - **C1:** Crescents present in <25% of glomeruli
  - **C2:** Crescents present in ≥25% of glomeruli ("Crescentic IgA Nephropathy", often requiring more aggressive immunosuppressive therapy)

**Clinical Significance:** Each component of the MEST-C score (especially T and C) is independently associated with the patient's long-term risk of developing kidney failure (uremia). The pathology report will provide the specific score (e.g., M1E0S1T0C0). Clinicians use this score in combination with the patient's clinical data (e.g., proteinuria level, blood pressure, eGFR) to comprehensively assess the condition and decide on a treatment plan.

#### ****2. Lee's Grading System - The Traditional Classic Standard****

This is an older grading system proposed in 1982, which divides IgA Nephropathy into Grades I-V. It was widely used in China for a long time because it is relatively intuitive. However, as it is more subjective, the distinction between grades is sometimes unclear, and its correlation with prognosis is less precise than the Oxford Classification, it is **now gradually being replaced by the Oxford Classification.** It is still mentioned in some historical literature and by older physicians.

- **Grade I:** Minimal changes, the vast majority of glomeruli are normal.
- **Grade II:** Mild changes, rare mesangial cell proliferation and sclerosis.
- **Grade III:** Focal and segmental glomerulonephritis, <50% of glomeruli show mesangial proliferation and sclerosis.
- **Grade IV:** Diffuse mesangial proliferative glomerulonephritis, 50%-100% of glomeruli show mesangial proliferation; crescents and sclerosis may be seen.
- **Grade V:** Diffuse sclerosing glomerulonephritis, >40% of glomeruli show global sclerosis, indicating advanced disease.

From the pathological diagnostic criteria, it can be known that diabetic nephropathy is not an immune disease and has nothing to do with the deposition of immune complexes. In contrast, IgA nephropathy is an immune disease, which is caused by the granular or mass deposition of immunoglobulins mainly composed of IgA in the glomerular mesangial area.
