# Supplementary material for: Trends, pathological classification of renal diseases proved by biopsy: A 10-year retrospective cohort study in an East Chinese Tertiary Center
Source: Medicine (Baltimore). 2026 May 8;105(19):e48595. doi: 10.1097/MD.0000000000048595 (PMC13166505; doi:10.1097/MD.0000000000048595)
Supplement: Supplementary file 2 [file medi-105-e48595-s002.docx]

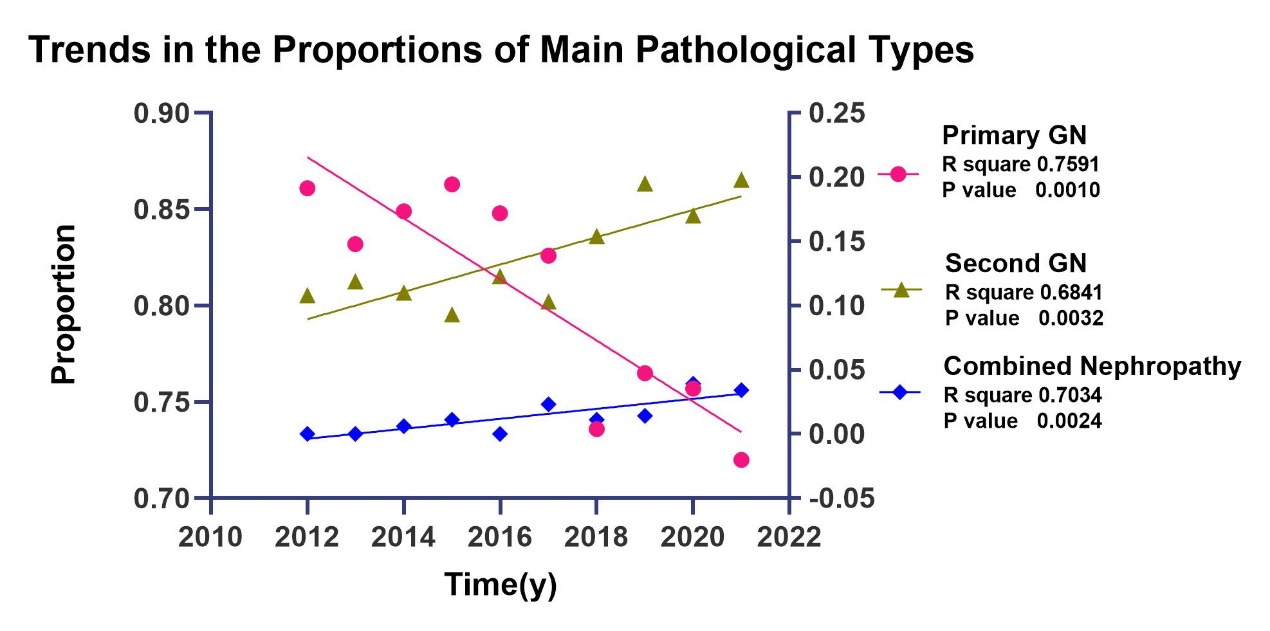


**Supplementary Figure 1.** Temporal trends in the proportions of Primary GN, Secondary GN, and combined nephropathy. Primary GN, primary glomerulonephritis; Secondary GN, secondary glomerulonephritis.
