# Supplementary figures and images for: Trends, pathological classification of renal diseases proved by biopsy: A 10-year retrospective cohort study in an East Chinese Tertiary Center
Source: Medicine (Baltimore). 2026 May 8;105(19):e48595. doi: 10.1097/MD.0000000000048595 (PMC13166505; doi:10.1097/MD.0000000000048595)

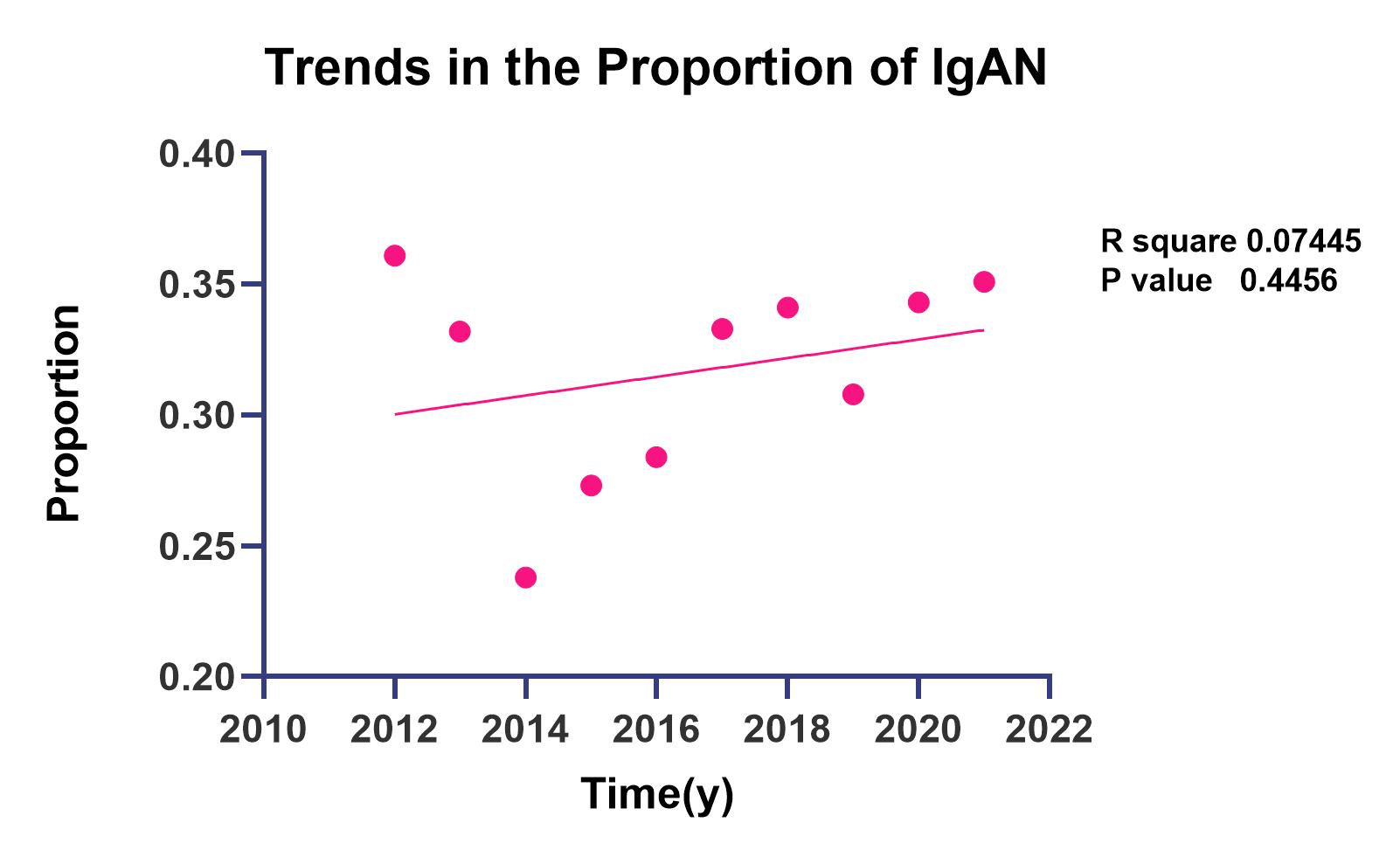


**Supplementary Figure 2.** Temporal trends in the proportions of IgAN. IgAN, IgA nephropathy.

Supplement: Supplementary file 3 [file medi-105-e48595-s003.docx]

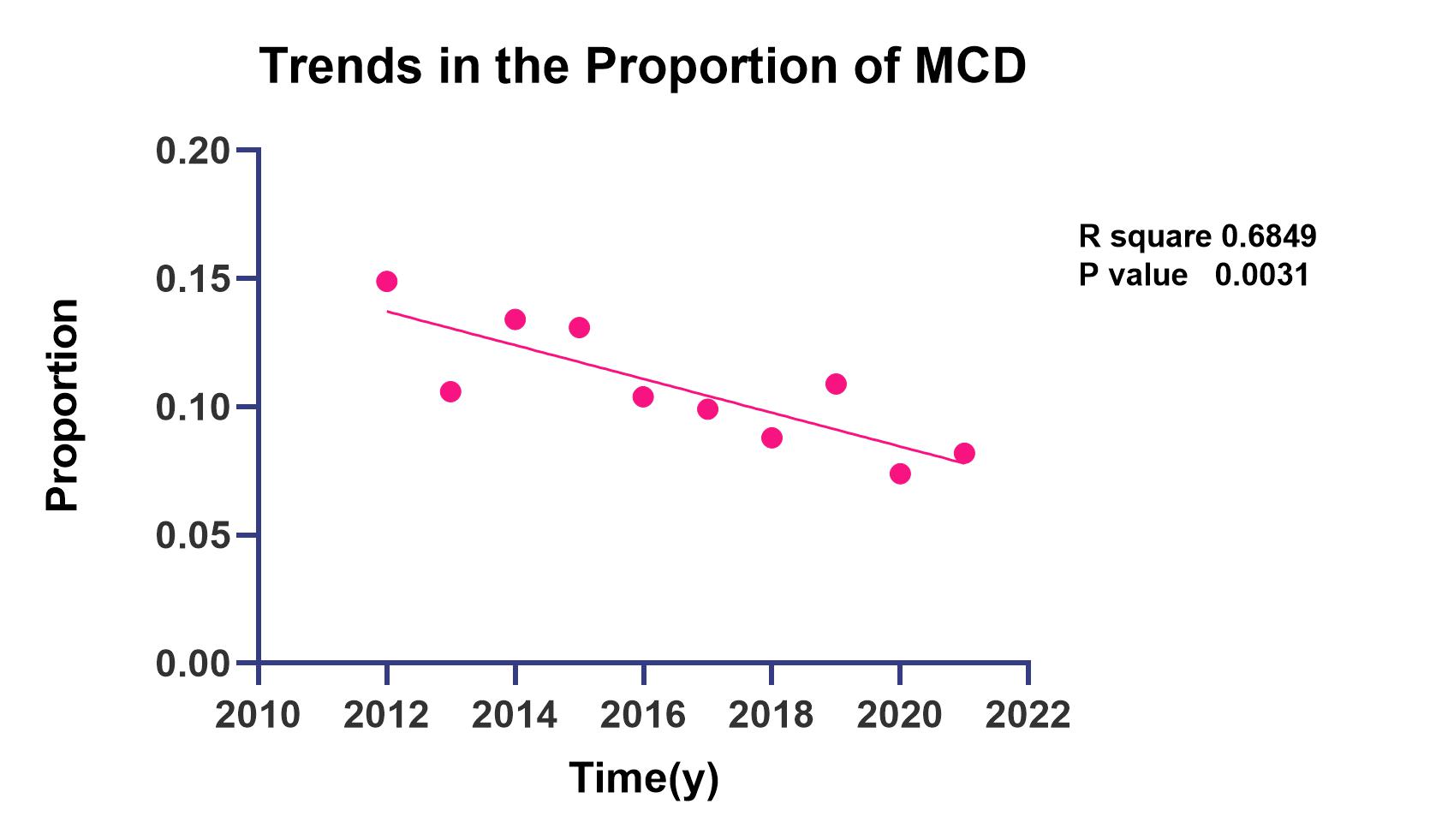


**Supplementary Figure 4.** Temporal trends in the proportions of MCD. MCD, minimal change disease.

Supplement: Supplementary file 5 [file medi-105-e48595-s005.docx]

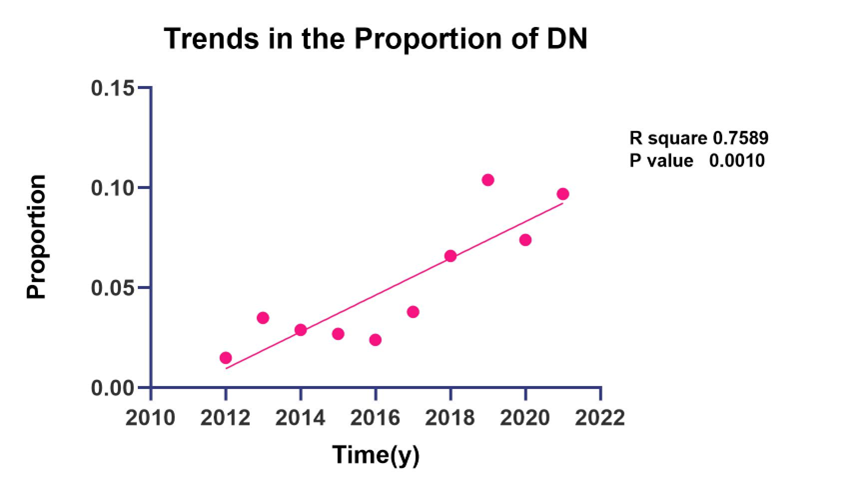


**Supplementary Figure 5.** Temporal trends in the proportions of DN. DN, diabetic nephropathy.

Supplement: Supplementary file 6 [file medi-105-e48595-s006.docx]

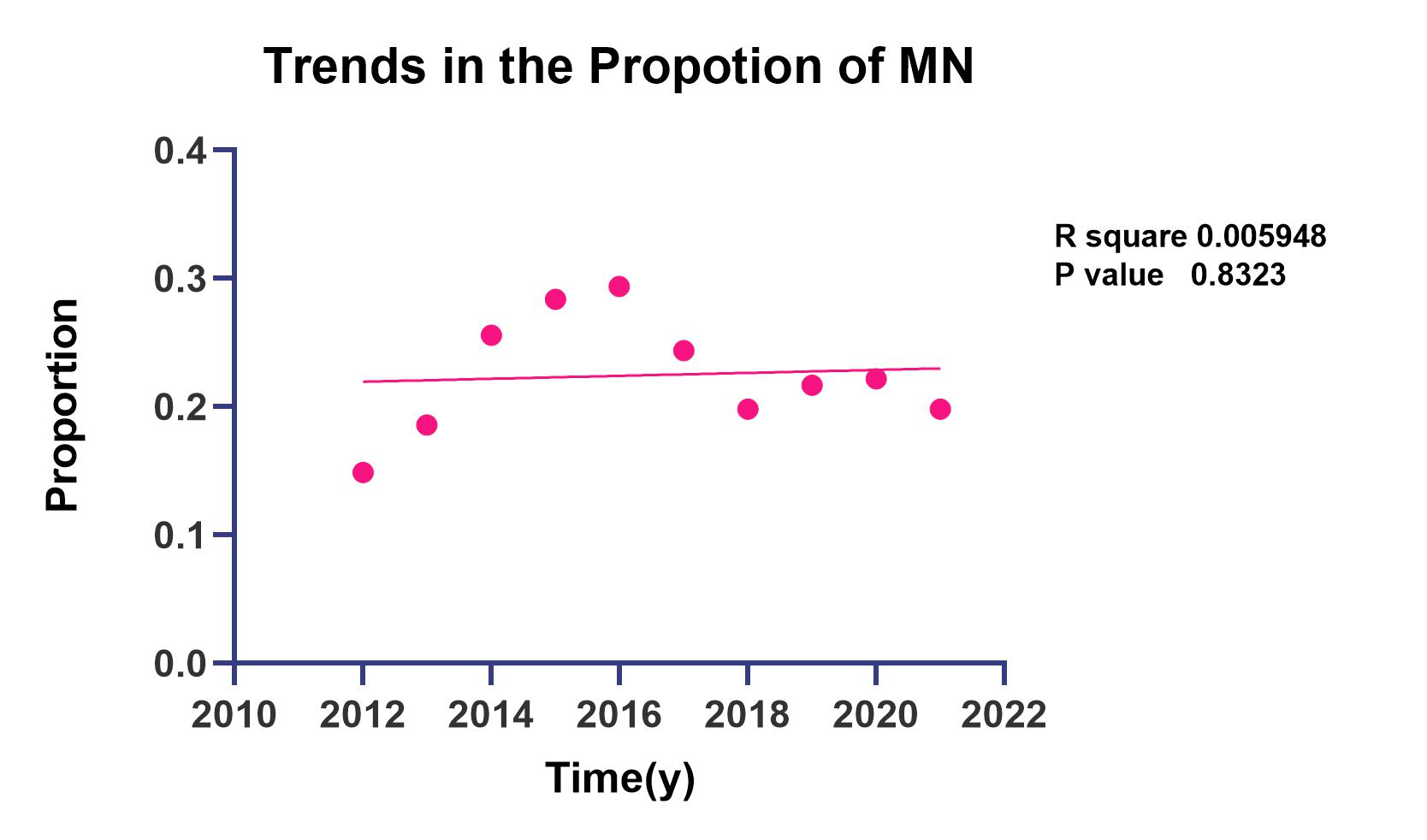


**Supplementary Figure 6.** Temporal trends in the proportions of MN. MN, membranous nephropathy.

Supplement: Supplementary file 7 [file medi-105-e48595-s007.docx]
